# Beam perturbation characteristics of a 2D transmission silicon diode array, Magic Plate

**DOI:** 10.1120/jacmp.v17i2.5932

**Published:** 2016-03-08

**Authors:** Ziyad A. Alrowaili, Michael L.F. Lerch, Marco Petasecca, Martin G. Carolan, Peter E. Metcalfe, Anatoly B. Rosenfeld

**Affiliations:** ^1^ Centre for Medical Radiation Physics University of Wollongong Australia; ^2^ Physics Department Aljouf University Aljouf Saudi Arabia; ^3^ Illawarra Cancer Care Centre Wollongong Hospital Wollongong Australia

**Keywords:** transmission detector, solid‐state 2D detector array, radiotherapy quality assurance, surface dose, dose reconstruction, *in vivo* dosimetry

## Abstract

The main objective of this study is to demonstrate the performance characteristics of the Magic Plate (MP) system when operated upstream of the patient in transmission mode (MPTM). The MPTM is an essential component of a real‐time QA system designed for operation during radiotherapy treatment. Of particular interest is a quantitative study into the influence of the MP on the radiation beam quality at several field sizes and linear accelerator potential differences. The impact is measured through beam perturbation effects such as changes in the skin dose and/or percentage depth dose (PDD) (both in and out of field). The MP was placed in the block tray of a Varian linac head operated at 6, 10 and 18 MV beam energy. To optimize the MPTM operational setup, two conditions were investigated and each setup was compared to the case where no MP is positioned in place (i.e., open field): (i) MPTM alone and (ii) MPTM with a thin passive contamination electron filter. The in‐field and out‐of‐field surface doses of a solid water phantom were investigated for both setups using a Markus plane parallel (Model N23343) and Attix parallel‐plate, MRI model 449 ionization chambers. In addition, the effect on the 2D dose distribution measured by the Delta^4^ QA system was also investigated. The transmission factor for both of these MPTM setups in the central axis was also investigated using a Farmer ionization chamber (Model 2571A) and an Attix ionization chamber. Measurements were performed for different irradiation field sizes of $5\times 5\;{\text{cm}}^{2}$ and $10\times 10\;{\text{cm}}^{2}$. The change in the surface dose relative to $d_{\text{max}}$ was measured to be less than 0.5% for the 6 MV, 10 MV, and 18 MV energy beams. Transmission factors measured for both set ups (i & ii above) with 6 MV, 10 MV, and 18 MV at a depth of $d_{\text{max}}$ and a depth of 10 cm were all within 1.6% of open field. The impact of both the bare MPTM and the MPTM with 1 mm buildup on 3D dose distribution in comparison to the open field investigated using the Delta^4^ system and both the MPTM versions passed standard clinical gamma analysis criteria. Two MPTM operational setups were studied and presented in this article. The results indicate that both versions may be suitable for the new real‐time megavoltage photon treatment delivery QA system under development. However, the bare MPTM appears to be slightly better suited of the two MP versions, as it minimally perturbs the radiation field and does not lead to any significant increase in skin dose to the patient.

PACS number(s): 87.50.up, 87.53.Bn, 87.55.N, 87.55.Qr, 87.56.Fc.

## I. INTRODUCTION

External photon beams such as intensity‐modulated radiation therapy (IMRT) and, more recently, volumetric‐modulated arc therapy (VMAT), which have highly conformal radiation delivery, have increasingly been applied for cancer treatment.^1^, ^2^ The main goal of these techniques is to achieve dose distribution of increased conformity to the target volumes, while further reducing the doses to the healthy normal tissue.^(3)^ VMAT consists of treating the patient by using one or more gantry arcs with continuously varying beam aperture shape, gantry speed, and dose rate.^4^, ^5^ The complexity of IMRT and VMAT in treatment delivery places new demands on ensuring the quality of the linear accelerator dose delivery in real time, as its controller must now determine in real time the gantry position (VMAT), the multileaf collimator (MLC) operation, and jaw speed, as well as the dose rate (VMAT & IMRT). The complexity of the systems involved requires new and appropriate methods and potentially new tools in order to do this. Several devices dedicated to daily QA have been developed that can be used for IMRT and VMAT verification.^6^, ^7^, ^8^, ^9^, ^10^, ^11^, ^12^

The 2D detector arrays based on ionization chambers play a major role in QA, but most of these are not suitable for *in vivo* 2D dose mapping.^10^, ^13^, ^14^, ^15^, ^16^, ^17^, ^18^ 2D semiconductor based on diodes approaches, such as MapCHECK (Sun Nuclear Corp., Melbourne, FL)^5^, ^11^, ^19^, ^20^, ^21^ and the quality control (QA) device,^(22)^ have also been used for dose verification. Electronic portal imaging devices (EPIDs) have also been tested for *in vivo* dosimetry purposes.^(12)^ In phantom, verification of the treatment plan in three dimensions (3D) can be carried out successfully using Delta^4^ (Scandidos AB, Uppsala, Sweden),^(23)^ ArcCHECK (Sun Nuclear Corp.),^5^, ^24^, ^25^, ^26^ and Octavius (PTW, Freiburg, Germany).^(27)^

There is considerable need for real‐time *in vivo* verification of the IMRT and the VMAT that can be achieved by a transmission‐type detector placed in the photon beam downstream of the MLC during treatment. The idea of a 2D array transmission detector for real‐time *in vivo* QA in external beam radiation therapy (EBRT) was first proposed by Paliwal et al.^(28)^ The transmission chamber was an off‐the‐shelf device called a Dose Area Product Meter (Gammex RMI, Inc., Middleton, WI)^(28)^ using the concept of dose‐area product to monitor the radiation beam in diagnostic applications for the measurement of patient exposure. The concept of a dose‐area product was further developed to allow online *in vivo* comparison of measured dose‐area product related to instantaneous MLC leaves opening and compare it with prerecorded data.^29^, ^30^

Most of the currently used transmission‐type detectors such as the COMPASS detector (IBA Dosimetry),^(31)^ the integral quality monitoring system (IQM),^(30)^ and the DAVID system^29^, ^32^ are based on pixelated ionization chambers and these systems have been shown to significantly increase the patient surface dose due to additional electron contamination. The presence of these transmission devices also causes beam attenuation up to 7% for a 6 MV photon beam for doses beyond $d_{\text{max}}$.^30^, ^31^, ^33^ Recently, wireless transmission detectors, such as Delta^4^ Discover (ScandiDos) based on a 2D diode array^(34)^ and Dolphin (IBA Dosimetry) based on a 2D ionization chamber array,^(35)^ have been introduced. These are very advanced *in vivo* QA dosimetry systems but clinical evaluation, and, in particular, their effect on skin dose and the perturbation of the radiation field still need to be evaluated. Although some of these QA delivery devices are available commercially, they are still rare in clinical practice for IMRT and VMAT delivery verification during patient daily treatment.

We are developing a solid‐state‐based detector system for real‐time QA during radiotherapy treatment, which is completely independent of the known linear accelerator operating conditions. The 2D silicon transmission diode array, called the Magic Plate (MP)^(36)^ is designed to be placed between the MLC and the patient, operating in transmission mode (MPTM). Therefore, the response of the 2D detector array can be correlated with the amount of 2D energy fluence incident on the patient and therefore with dose. Recently we have demonstrated possibility of 2D dose reconstruction at different depths in a phantom for different field configurations based on MPTM response.^(37)^ The QA system will therefore provide immediate real‐time feedback simultaneously with the patient treatment, leading to a very robust QA in treatment delivery.

The Magic Plate has been previously reported in dose mode (MPDM) to map a 2D dose distribution in a phantom.^(36)^ However, for the real‐time QA system, the MP is placed in the accessory slot on the head of the linac and must be operated as a transmission detector during the patient treatment with minimal perturbation of the incident radiation field, requiring a detailed study of its influence on the treatment beam. The surface dose in particular can be one of the limiting factors in treatment plans with high doses to a target and is the focus of this work.^(38)^ Similar studies in the past have been completed for beam modifiers such as wedges, MLC, and block trays.^39^, ^40^, ^41^, ^42^ Beam perturbation can be quantified by measuring changes in the percentage depth dose (PDD) profiles particularly in the buildup region, including surface dose, with the transmission MP placed in the beam. Ideally, such a device would have no significant impact on the treatment delivery, either to the target dose or surrounding normal tissues. Therefore, the main objective of this study is to quantitatively determine whether or not the presence of the MP upstream of the patient has a significant effect on the surface dose and PDD during patient treatment.

## II. MATERIALS AND METHODS

**Figure 1:**
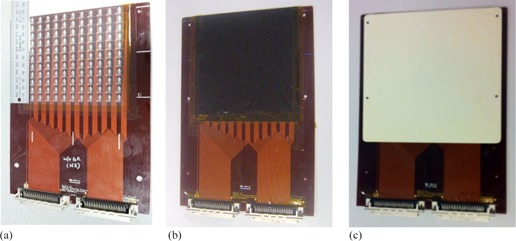
Magic Plate 2D diode array is shown. Magic Plate (MP) (a), designed at CMRP, University of Wollongong, with two possible setup conditions: (b) the bare MP array (sandwiched between black 80 μm thick plastic sheets), and (c) the bare MP array (covered by 1 mm solid water through entire MP).

The Magic Plate (MP) is a 2D detector array of 11×11 silicon epitaxial diodes covering an area of $10\times 10\;{\text{cm}}^{2}$ with a thickness of 0.45 mm, as shown in Fig. 1(a). The physical size of a single epidiode is $1.5\times 1.5\times 0.425\;{\text{mm}}^{3}$. The MP is designed to be operated in both transmission mode (MPTM) and dose mode (MPDM), and details of the MP design, preliminary characterization and the individual epitaxial silicon diodes utilized have been published elsewhere.^36^, ^43^ For the MPTM investigation we examined two potential operational setup conditions:

(i) The MP array sandwiched between black plastic sheets, 80 μm thick, here in referred as “bare MPTM” as shown in Fig. 1(b). The role of the black plastic is to remove light from the background detector response.
(ii) The MP array with an additional 1 mm of solid water covering the entire array as shown in Fig. 1(c). The role of the 1 mm solid water was to remove scattered low‐energy electrons.

Condition (i) is designed for minimum attenuation of the primary linac beam; however, precise sampling of the energy fluence may be adversely affected by contamination electrons. To better sample the energy fluence the 1 mm of solid water was introduced in condition (ii) to filter a large component of these contamination electrons. The surface dose and percentage depth dose (PDD) for each setup (i.e., (i) and (ii) above) was measured and compared to the corresponding surface dose and PDD with no MP in place (i.e., open field). These measurements were carried out both in‐field (on the beam central axis (CAX)) and out‐of‐field (OOF) for different field sizes.

Experiments were performed on two Varian linear accelerators (Model 2100EX), one of which operated at the energies of 6 MV and 10 MV, and another which was operated at 18 MV. Irradiation field sizes of $5\times 5\;{\text{cm}}^{2}$ and $10\times 10\;{\text{cm}}^{2}$ and a constant SSD of 100 cm were used for both linac experiments. Ionization chamber measurements were normalized to the maximum dose at a depth of 15 mm for 6 MV, 21 mm for 10 MV, and 30 mm for 18 MV beam energies. With the MPTM mounted on the linac accessory slot, PDD up to a depth of 10 cm were measured on the central axis (CAX) and out of the field (OOF) 11 cm laterally to CAX as a function of the irradiation field size. For the OOF measurements, the Solid Water phantom (Gammex RMI, Middleton, WI) and detector on the linac patient couch were moving laterally from the central axis position. The phantom size was $60\times 30\times 30\;{\text{cm}}^{3}$ with the lateral movement in the direction of the 60 cm phantom length to maintain consistent scattering conditions.

The depth‐dose profile and surface dose were measured with Markus type (PTW, Freiburg, Germany) parallel plate ionization chamber Model N23343. The chamber was connected via a tri‐axial cable to a PTW UNIDOS model T10002‐20713 electrometer with −300 V bias voltage applied. The Markus chamber is designed for the measurement of surface and buildup dose. Markus‐type chambers are known for their overresponse due to the large separation and their small guard ring,^44^, ^45^ and different methods have been developed to correct for this overresponse.^46^, ^47^, ^48^, ^49^ In this study, all measurements using the Markus ionization chamber were corrected using the Velkley correction as modified by Rawlinson to correct the overresponse of the Markus chamber. The chamber dimension used for the correction calculation was obtained from Chen et al.^(50)^ A dose of 200 MU was used for all measurements.

**Figure 2:**
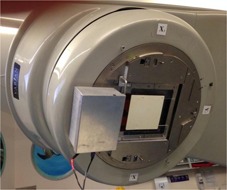
Transmission mode Magic Plate setup. MPTM, just below the linac jaw of the medical linear accelerator with a prototype version of the MP readout system on the head of a Varian linac.

All measurements were performed with MPTM (source‐to‐detector distance (SDD) is 58 cm) for the two different setup conditions mentioned above and without MP (open field) as shown in Fig. 2. All measurements were repeated at least three times in order to calculate the average value and standard deviation.

The depth dose as a function of field size in the solid water phantom were carried out using the Markus chamber mentioned above with some data compared with that using an Attix chamber (Gammex RMI), RMI model 449 for the same measurements.

In the second part of this study, the influence of both the bare MPTM and the MPTM with 1 mm buildup on the open field 3D dose distribution (measured change as a percentage) was measured by the Delta^4^ (Scandidos AB) QA system.

Different treatment field configurations were used using the Delta^4^ system positioned at isocenter for both MPTM versions to study Delta^4^ response in comparison with open field. A 6 MV photon beam and different treatment configurations were used for both MPTM versions (bare MPTM and MPTM with 1 mm buildup). The center of the Delta^4^ phantom was aligned at isocenter using the relevant markings on the PMMA. A daily correction factor (DCF) was applied to Delta^4^ system measurements in order to account for differences in treatment conditions between the time of calibration and time of measurement. The DCF is calculated for a uniform, square field $20\times 20\;{\text{cm}}^{2}$. The relevant DCF has been applied to all measurements. Prior to calculation of the DCF, the temperature is measured on the surface of the electrometer unit. The correction is calculated relative to the temperature at the time of absolute calibration. The temperature is input to the software and a correction is applied internally.

In the third part of this work, we investigated the transmission factor of the MP. The transmission factor was derived from any dose change measured at $d_{\text{max}}$ and at a depth of 10 cm for radiation field sizes $10\times 10\;{\text{cm}}^{2}$ and $5\times 5\;{\text{cm}}^{2}$ and an SSD of 100 cm for 6 MV, 10 MV, and 18 MV photon fields. A Farmer ionization chamber (Model 2571A) was used for 6 MV, 10 MV, and an Attix chamber, Model 449, was used for the 18 MV. Delta^4^ system also was used to measure the transmission characteristics for 6 MV photon beam using different treatment configurations for both MP versions in comparison with the open field configuration.

## III. RESULTS

**Figure 3:**
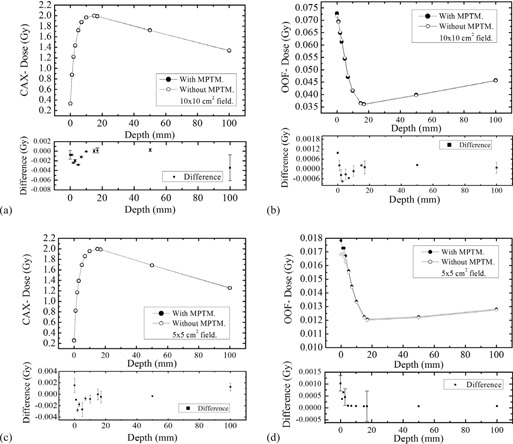
Depth‐dose measurements at 6 MV. Depth dose and dose difference distributions in a solid water phantom for a 6 MV linac field, SSD 100 cm, with and without MPTM, for different field sizes on the CAX: (a) $10\times 10\;{\text{cm}}^{2}$, (c) $5\times 5\;{\text{cm}}^{2}$; and OOF field sizes (b) $10\times 10\;{\text{cm}}^{2}$, (d) $5\times 5\;{\text{cm}}^{2}$. The solid circles indicate that the response of ionization chamber when MP was operated in transmission mode, and the open circles indicate the response of the ionization chamber without MP in transmission mode.

Figure 3 shows the depth dose profile of a 6 MV photon beam on the CAX and OOF for a $10\times 10\;{\text{cm}}^{2}$ and $5\times 5\;{\text{cm}}^{2}$ field size, respectively, with the bare MPTM (setup (i)) mounted in the linac accessory. Error bars (where visible) represent 3 SDs from the mean. The square symbols in each graph show the measured change (plotted separately) in the depth‐dose curve (DDC) caused by the MPTM in the beam.

Figures 3(a) and (c) demonstrate that the perturbation effect of the radiation beam by MPTM is negligible. The change in the surface dose with the MPTM placed in the beam was a reduction of 0.0751 cGy (0.2270%) for $10\times 10\;{\text{cm}}^{2}$ and an increase of 0.1553 cGy (0.6125%) for $5\times 5\;{\text{cm}}^{2}$ field sizes. For all depths investigated, the maximum measured change in the absolute dose was a reduction of less than 0.2839 cGy (0.1647%) for $10\times 10\;{\text{cm}}^{2}$ and 0.2769 cGy (0.2363%) for $5\times 5\;{\text{cm}}^{2}$ field sizes. The majority of the change occurs within 5 mm of the surface. The measured changes in the CAX dose at any depths with MPTM in a beam were less than 1% of the equivalent open field value. The similar experiments on the 6 MV beam using the MPTM with additional 1 mm buildup showed an increase of the surface dose 0.9776 cGy (2.9554%) for $10\times 10\;{\text{cm}}^{2}$ and 0.1001 cGy (0.3910%) for $5\times 5\;{\text{cm}}^{2}$ field sizes.

In the case of the OOF, the effect of the MPTM on radiation field perturbation is greater in terms of percentage change than for the equivalent CAX measurements. In addition, the majority of the change occurs within 10 mm below the phantom surface. With MPTM placed in the beam, the surface dose increased by 0.1076 cGy (1.4997%) for the $10\times 10\;{\text{cm}}^{2}$ and 0. 1051 cGy (6.2561%) $5\times 5\;{\text{cm}}^{2}$ field sizes, as shown in Figs. 3(b) and (d). The equivalent using the MPTM with an additional 1 mm solid water buildup showed 0.8031 cGy (10.8580%) for $10\times 10\;{\text{cm}}^{2}$ and 0. 2914 cGy (16.9675%) for $5\times 5\;{\text{cm}}^{2}$ field sizes.

**Figure 4:**
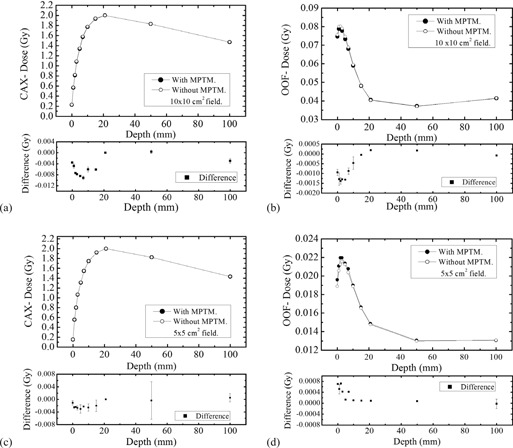
Depth‐dose measurements at 10 MV. Depth dose and dose difference distributions in a solid water phantom for 10 MV linac field, SSD 100 cm, with and without MPTM, for different field sizes on the CAX: (a) $10\times 10\;{\text{cm}}^{2}$, (c) $5\times 5\;{\text{cm}}^{2}$; and OOF field sizes (b) $10\times 10\;{\text{cm}}^{2}$, (d) $5\times 5\;{\text{cm}}^{2}$. The solid circles indicate that the response of ionization chamber when MP was operated in transmission mode, and the open circles indicate the response of the ionization chamber without MP in transmission mode.

Figure 4 shows the depth‐dose profile of the 10 MV photon beam on the CAX and OOF for $10\times 10\;{\text{cm}}^{2}$ and $5\times 5\;{\text{cm}}^{2}$ field sizes with the bare MPTM (setup (i)) mounted in the linac accessory.

Similar to the 6 MV X‐ray beam, the perturbation effect of the bare MPTM is small. The change in the surface dose by the introduction of the MPTM was a reduction of 0.3576 cGy (1.5604%) for $10\times 10\;{\text{cm}}^{2}$ and 0. 1097 cGy (0.7140%) for $5\times 5\;{\text{cm}}^{2}$ field sizes.

From Figs. 4(a) and (c) one can see that, for all depths investigated, the maximum measured change in absolute dose was less than −0.9138 cGy (−0.5778%) and −0.3045 cGy (−0.2322%) for the $10\times 10\;{\text{cm}}^{2}$ and $5\times 5\;{\text{cm}}^{2}$ field sizes, respectively. The greatest dose change occurs within 7 mm of the surface. The perturbation effect of MPTM on the depth‐dose change in the CAX is less than 1% for all measured depths. A similar experiment with 1 mm solid water buildup placed above the MPTM showed an increase of the surface dose by +0.3280 cGy (+1.4345%) and +0.0552 cGy (+0.3576%) for $10\times 10\;{\text{cm}}^{2}$ and $5\times 5\;{\text{cm}}^{2}$ field sizes, respectively.

In OOF, the perturbation effect of the MPTM on depth‐dose distribution is slightly higher than for the same dose in a CAX. Most dose change is within 10 mm from the surface. The surface dose with MPTM in the beam decreased slightly −0.0938 cGy (−1.2412%) for $10\;\text{cm}\times 10\;{\text{cm}}^{2}$ and increased +0.0702 cGy (+3.7167%) for the $5\times 5\;{\text{cm}}^{2}$ field sizes. The equivalent experiments using the MPTM with an additional 1 mm buildup showed an increase of the surface dose +0.5858 cGy (+7.5330%) and +0.3749 cGy (+20.2024%) for $10\times 10\;{\text{cm}}^{2}$ and $5\times 5\;{\text{cm}}^{2}$ field sizes, respectively.

**Figure 5:**
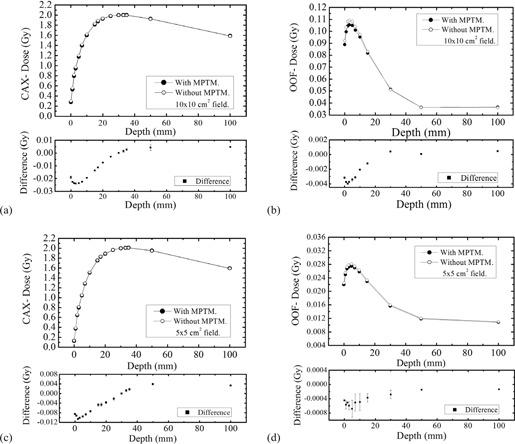
Depth‐dose measurements at 18 MV. Depth dose and dose difference distributions in a solid water phantom for 18 MV linac field, SSD 100 cm, with and without MPTM, for different field sizes in CAX: (a) $10\times 10\;{\text{cm}}^{2}$, (c) $5\times 5\;{\text{cm}}^{2}$; and OOF field sizes (b) $10\times 10\;{\text{cm}}^{2}$, (d) $5\times 5\;{\text{cm}}^{2}$. The solid circles indicate that the response of ionization chamber when MP was operated in transmission mode, and the open circles indicate the response of the ionization chamber without MP in transmission mode.

Figure 5 shows the depth‐dose profile of an 18 MV photoneutron beam on the CAX and OOF for $10\times 10\;{\text{cm}}^{2}$ and $5\times 5\;{\text{cm}}^{2}$ field sizes with the bare MPTM (setup (i)) mounted in the linac accessory.

As for 6 MV and 10 MV X‐ray beams, the perturbation effect of the bare MPTM is small. The change in the surface dose by the MPTM was −1.8991 cGy (−6.3814%) for $10\times 10\;{\text{cm}}^{2}$ and −0.8535 cGy (−6.2781%) for $5\times 5\;{\text{cm}}^{2}$ field sizes.

From Figs. 5(a) and (c) one can see that, for all depths investigated, the maximum measured change in absolute dose was less than +0.4773 cGy (+0.3013%) and +0.3947 cGy (+0.2025%) for the $10\times 10\;{\text{cm}}^{2}$ and $5\times 5\;{\text{cm}}^{2}$ field sizes, respectively. The most dose change occurs within 10 mm from the surface. The perturbation effect of MPTM on depth‐dose change in the CAX is less than 1% for all measured depths. A similar experiment with 1 mm solid water buildup placed above the MPTM showed an increase of surface dose of −2.4356 cGy (−8.1842%) and −1.0862 cGy (−7.9895%) for $10\times 10\;{\text{cm}}^{2}$ and $5\times 5\;{\text{cm}}^{2}$ field sizes, respectively.

In OOF, the perturbation effect of the MPTM on depth‐dose distribution is slightly higher than in a field. Most dose change is within 10 mm of the surface. The surface dose with MPTM in the beam slightly decreased by 0.3169 cGy (−3.4432%) for $10\times 10\;{\text{cm}}^{2}$ and decreased by 0.04476 cGy (−1.9931%) for the $5\times 5\;{\text{cm}}^{2}$ field sizes. The equivalent experiments using the MPTM with additional 1 mm buildup showed a decrease in the surface dose of 0.1804 cGy (−1.9631%) and an increase of 0.08228 cGy (+3.6623%) for $10\times 10\;{\text{cm}}^{2}$ and $5\times 5\;{\text{cm}}^{2}$ field size, respectively.

Table 1 summarizes the findings of the beam perturbation by MPTM at $d_{\text{max}}$ for all the above‐presented radiation fields.

The quantitative absolute response (in nC per 100 MU) of the bare MPTM and the MPTM array with the additional 1 mm of solid water were measured for a $10\times 10\;{\text{cm}}^{2}$ and $5\times 5\;{\text{cm}}^{2}$ field sizes and beam energies of 6 MV, 10 MV, and 18 MV. The data are presented in Table 2. As expected, 1 mm buildup is increasing the response of the diodes, however the signal is enough in both cases to reliably measure signal with accuracy better than 0.1% with developed 512 channel electrometer.

**Figure 6:**
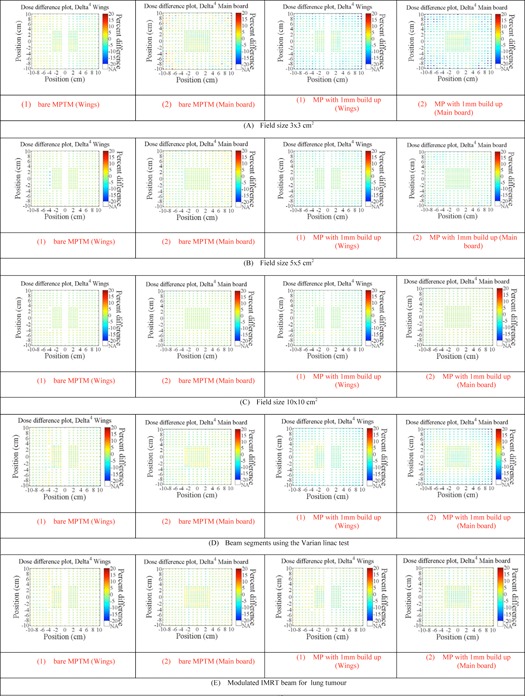
Impact on the measured dose (in percentage difference) by Delta^4^ phantom for a variety of treatment field configurations. All data shown are with MP in transmission mode (bare MPTM or MPTM with 1 mm buildup) comparison with no MP in place.

Figure 6 shows the effect of having the MPTM system in place on the measured 2D dose mapping by the Delta^4^ phantom diode array for a range of treatment field configurations. All data presented here are with the MP in transmission mode (the bare MPTM or MPTM with 1 mm buildup) with the data compared to the open field configuration with no MPTM in place. The measured changes in percent difference across the entire Delta^4^ detectors have been displayed in Fig. 6. In the case of the bare MPTM, the measured change in‐field in percent difference (2 SDs) were ±3.24%, ±2.77%, ±1.97%, ±2.75%, and ±2.72% for the $3\times 3\;{\text{cm}}^{2}$, $5\times 5\;{\text{cm}}^{2}$, $10\times 10\;{\text{cm}}^{2}$, the beam segments, and modulated IMRT field configurations, respectively, for 6 MV photon beam. A similar experiment with 1 mm solid water buildup placed above the MPTM showed that the in‐field measured change in percent difference (2 SDs) were ±3.47%, ±0.62%, ±2.05%, ±3.39%, and ±3.10% for the $3\times 3\;{\text{cm}}^{2}$, $5\times 5\;{\text{cm}}^{2}$, $10\times 10\;{\text{cm}}^{2}$, the beam segments, and modulated IMRT beam configurations, respectively, for 6 MV photon beam.

**Table 1:** Summary of the percentage depth dose (PDD) measurements with different energies

		*Original Markus Ionization Chamber*				*Attix Ionization Chamber*			
		*CAX (%)*		*OOF (%)*		*CAX (%)*		*OOF (%)*	
*Energy (MV)*	*Field Size (cm^2^)*	*MP field no buildup*	*MP field with 1 mm buildup*	*MP field no buildup*	*MP field with 1 mm buildup*	*MP field no buildup*	*MP field with 1 mm buildup*	*MP field no buildup*	*MP field with 1 mm buildup*
6	10×10	−0.0375	0.4888	0.0538	0.4015	0.0767	0.4610	0.0485	0.3563
	5×5	0.0776	0.0500	0.0525	0.1457	0.0157	0.1505	0.0312	0.1473
10	10×10	−0.1780	0.1640	−0.0469	0.2929	−0.2559	0.1444	−0.0483	0.2428
	5×5	−0.0548	0.0276	0.0351	0.1874	−0.0720	0.0527	0.0221	0.1195
18	10×10	...	...	...	...	−0.9495	−1.2178	−0.1584	−0.0902
	5×5	...	...	...	...	−0.4267	−0.5431	−0.0223	0.0411

**Table 2:** Summary of the response (sensitivity) (2 SDs) of the MP in transmission mode (MPTM)

*MPTM Central Diode Response (nC)*			
*Energy (MV)*	*Field Size (cm^2^)*	*MP field no buildup*	*MP field with 1 mm buildup*
6	10×10	68.0281	72.4019
		(±0.0257)	(±0.1270)
	5×5	57.0172	65.3595
		(±0.2208)	(±0.0253)
10	10×10	55.0714	73.5076
	5×5	39.1567	59.1128
		(±0.0053)	(±0.0056)
18	10×10	53.8847	61.9483
		(±0.2306)	(±0.1526)
	5×5	34.7484	46.4496
		(±0.0304)	(±0.0776)

The absolute response (in nano‐Coulombs per 100 MU) of the central detector (CAX) of the MPTM located on the central axis of the 6 MV, 10 MV, and 18 MV radiation fields, as a function of field size.

Gamma analysis was performed using 3% and 3 mm criteria. By comparing the planned dose to the delivered dose, a plan will fail the criteria if <90% of all data points have a gamma index ≤1. Gamma analysis criteria (±3% dose deviation, ≤3 mm distance to agreement and ≤1 gamma index) for both MPTM setups (i and ii) and open field for all configurations of delivered radiation fields was carried out based on comparison of 3D doses reconstructed by the Delta^4^ system in comparison with 3D dose predicted by treatment planning system (TPS). The gamma index gave 100% (90%) pass criteria for all treatment configurations. Hence, this gamma threshold was not sensitive to the inclusion of the bare MPTM or MPTM with 1 mm buildup.

The transmission factor related to the dose change at $d_{\text{max}}$ and 10 cm depth in a solid water phantom for two field sizes ($10\times 10\;{\text{cm}}^{2}$ and $5\times 5\;{\text{cm}}^{2}$) for 6 MV, 10 MV, and 18 MV energy beams were measured and are presented in Table 3. Transmission factors at $d_{\text{max}}$ and a depth of 10 cm were all within 1.630% of open field. This increases the rigidity and potential lifetime reliability of the MP system. In the case of transmission detectors, both the IQM system and the DAVID system attenuate a 6 MV photon beam by 7%, while the COMPASS system reported a 3.3% beam attenuation, which are both greater than the values measured for the MP system. The recently introduced ScandiDos Delta4 Discover system attenuates the beam by up to 1%,^(34)^ whereas no data have yet been reported for the IBA Dolphin detector.

The transmission characteristics for 6 MV photon beam using different treatment configurations for both MPTM versions in comparison with the open field configuration was measured by Delta^4^ system at isocenter by finding difference in signals measured by all corresponding diodes in a field. In the case of the bare MPTM, the measured in‐field transmission factors (2 SDs) were 0.9902 (±3.24%), 0.9931 (±2.76%), 0.9950 (±1.96%), 0.9968 (±2.74%), and 0.9984 (±2.72%) for the $3\times 3\;{\text{cm}}^{2}$, $5\times 5\;{\text{cm}}^{2}$, $10\times 10\;{\text{cm}}^{2}$, the beam segments, and modulated IMRT field configurations, respectively, for 6 MV photon beam. A similar experiment with 1 mm solid water buildup placed above the MPTM showed that the in‐field measured transmission factors (2 SDs) were 0.9835 (±3.46%), 0.9829 (±0.62%), 0.9850 (±2.04%), 0.9863 (±3.38%), and 0.9873 (±3.1%) for the $3\times 3\;{\text{cm}}^{2}$, $5\times 5\;{\text{cm}}^{2}$, $10\times 10\;{\text{cm}}^{2}$, the beam segments, and modulated IMRT beam configurations, respectively, for 6 MV photon beam. These transmission characteristics measured are similar to those measured by an ionization chamber, as quoted in Table 3.

**Table 3:** The transmission factor (2 SDs) at depth of $d_{\text{max}}$ and at depth of 10 cm for 6 MV, 10 MV, and 18 MV energy beams

		*At Depth of Maximum Dose*		*At Depth of 10 cm*	
*Energy (MV)*	*Field Size (cm^2^)*	*MP field no buildup*	*MP field with 1 mm buildup*	*MP field no buildup*	*MP field with 1 mm buildup*
6	10×10	0.9937	0.9841	0.9944	0.9855
		(±0.0026)	(±0.0021)	(±0.0068)	(±0.0028)
	5×5	0.9951	0.9853	0.9967	0.9845
		(±0.0028)	(±0.0017)	(±0.0030)	(±0.0019)
10	10×10	0.9944	0.9863	0.9949	0.9867
		(±0.0012)	(±0.0022)	(±0.0003)	(>±0.0000)
	5×5	0.9948	0.9865	0.9951	0.9865
		(±0.0010)	(±0.0006)	(±0.0005)	(±0.0006)
18	10×10	0.9930	0.9856	0.9959	0.9901
		(±0.0006)	(±0.0007)	(±0.0002)	(±0.0001)
	5×5	0.9943	0.9902	0.9964	0.9853
		(±0.0004)	(±0.0006)	(±0.0002)	(±0.0004)

The Farmer ionization chamber was used for 6 MV and 10 MV and the Attix chamber was used for 18 MV.

## IV. DISCUSSION

Two potential operational setup conditions of the MPTM were considered to investigate the perturbation of the radiation field, the first with a Magic Plate covered by a thin 80 μm layer of light protection plastic and the second with an additional 1 mm solid water buildup on top of the MPTM. The 1 mm solid water buildup was introduced to reduce the sensitivity of the MPTM response to contamination electrons from the source and scattered from the jaws, thus making correlation with the energy fluence less challenging.

Results suggest that for the majority of the radiation fields studied, the electrons produced from the interaction of the 6 MV and the 10 MV photons with the jaws are scattered. Most of these electrons are peaked forward and located close to the jaw surfaces, minimizing any contribution to the center of the $10\times 10\;{\text{cm}}^{2}$ field at the phantom surface. This is supported by the increase in the relative surface dose when adding a 1 mm solid water slab above the MP which acts as a buildup for photons and an attenuator of scattered low‐energy electrons. However, in the case of a $5\times 5\;{\text{cm}}^{2}$ field, the role of electrons scattered from the jaw to the center of the field is essential. This is supported by a small change in the relative surface dose caused by the addition of the 1 mm slab above the MP. This effect is a combination of the attenuation of the low‐energy electrons scattered from the jaws and the generation of electrons from direct photon interactions within the 1 mm solid water buildup.

It was demonstrated that the OOF surface dose enhancement produced by a 1 mm buildup was greater than when the bare MPTM was used. This result is consistent with the CAX measurements and supports the radiation field perturbation interpretations on the CAX. The CAX and OOF dose with depth with the bare MPTM (Figs. 3 to 5) in place shows that the MPTM has negligible effect otherwise for all investigated fields and photon energies. The depth‐dose distribution perturbation is less than 1% at any depth, less than 10 cm in solid water. Perturbation factors at $d_{\text{max}}$ and at a depth of 10 cm were found to be less than 1.63% of the open field for all energies tested. The MPTM therefore produces very minimal distortion of the field and skin dose, both in field and OOF. Monte Carlo simulations are required for detailed explanation of the observed dose reduction and enhancement due to the MPTM which will form part of future work.

## V. CONCLUSIONS

The impact of the black plastic sheet and the 1 mm of solid water on the changes in the skin dose and full PDD have been investigated. The results showed that in general, both versions of the MPTM detector array could potentially be used as a 2D transmission detector. However, if the surface dose considered as an important factor to be considered for the cancer treatment, then the bare MPTM proved to be the best candidate as the increase to the surface dose was the least of the two systems tested.

The design of the MP (detectors and packaging) has been shown to be suitable for mounting the full QA device in the linac accessory slot during patient radiotherapy treatment. We have demonstrated that at 6 MV, 10 MV, and 18 MV the MPTM in the beam minimally perturbs the radiation field both in a field and out of field, leading to a <0.5% increase in dose at the surface relative to dose at $d_{\text{max}}$ and <1% in depth of the phantom. This change is minimal and within any dosimetry budgeted error, while potentially allowing for real‐time verification of VMAT and IMRT during patient treatment. Further work will be directed to the conversion of the response of MPTM directly to the absorbed dose in a patient without using the beam model.

## ACKNOWLEDGMENTS

Ziyad Awadh Alrowaili was supported by the Ministry of Education, Aljouf University, Kingdom of Saudi Arabia. The authors would like to acknowledge the National Health & Medical Research Council of Australia (NHMRC) which funded this project by the Project Grant No. 1029432.

## COPYRIGHT

This work is licensed under a Creative Commons Attribution 4.0 International License.

## Supporting information

Supplementary Material Files

Supplementary Material Files

Supplementary Material Files

Supplementary Material Files

Supplementary Material Files

Supplementary Material Files

Supplementary Material Files

Supplementary Material Files

Supplementary Material Files

Supplementary Material Files

Supplementary Material Files

Supplementary Material Files

Supplementary Material Files

Supplementary Material Files

Supplementary Material Files

Supplementary Material Files

Supplementary Material Files

Supplementary Material Files

Supplementary Material Files

Supplementary Material Files

Supplementary Material Files

Supplementary Material Files

Supplementary Material Files

Supplementary Material Files

Supplementary Material Files

Supplementary Material Files

Supplementary Material Files

Supplementary Material Files

Supplementary Material Files

Supplementary Material Files

Supplementary Material Files

Supplementary Material Files

Supplementary Material Files

Supplementary Material Files

Supplementary Material Files

Supplementary Material Files

Supplementary Material Files

Supplementary Material Files

Supplementary Material Files

Supplementary Material Files

Supplementary Material Files

Supplementary Material Files

Supplementary Material Files

Supplementary Material Files

Supplementary Material Files

Supplementary Material Files

Supplementary Material Files

Supplementary Material Files

Supplementary Material Files

Supplementary Material Files

Supplementary Material Files

Supplementary Material Files

Supplementary Material Files

## References

[1] Stieler F , Wolff D , Schmid H , Welzel G , Wenz F , Lohr F . A comparison of several modulated radiotherapy techniques for head and neck cancer and dosimetric validation of VMAT. Radiother Oncol. 2011;101(3):388–93. 21962821 10.1016/j.radonc.2011.08.023

[2] Khan FM . The physics of radiation therapy. Baltimore, MD: Lippincott Williams & Wilkins; 2009.

[3] Mijnheer B , Beddar S , Izewska J , Reft C . In vivo dosimetry in external beam radiotherapy. Med Phys. 2013;40(7):070903. 23822404 10.1118/1.4811216

[4] Otto K . Volumetric modulated arc therapy: IMRT in a single gantry arc. Med Phys. 2008;35(1):310–17. 18293586 10.1118/1.2818738

[5] Feygelman V, Nelms BE, editors. Dose Verification in IMRT and VMAT. AIP Conference Proceedings. 2011;1345(1).

[6] Qi Z‐Y , Deng X‐W , Huang S‐M , et al. Real‐time in vivo dosimetry with MOSFET detectors in serial tomotherapy for head and neck cancer patients. Int J Radiat Oncol Biol Phys. 2011;80(5):1581–88. 21237583 10.1016/j.ijrobp.2010.10.063

[7] Mijnheer B . State of the art of in vivo dosimetry. Radiat Prot Dosimetry. 2008;131(1):117–22. 18765403 10.1093/rpd/ncn231

[8] Wendling M , Louwe RJW , McDermott LN , Sonke JJ , Van Herk M , Mijnheer BJ . Accurate two‐dimensional IMRT verification using a back‐projection EPID dosimetry method. Med Phys. 2006;33(2):259–73. 16532930 10.1118/1.2147744

[9] Essers M and Mijnheer BJ . In vivo dosimetry during external photon beam radiotherapy. Int J Radiat Oncol Biol Phys. 1999;43(2):245–59. 10030247 10.1016/s0360-3016(98)00341-1

[10] Stasi M , Giordanengo S , Cirio R , et al. D‐IMRT verification with a 2D pixel ionization chamber: dosimetric and clinical results in head and neck cancer. Phys Med Biol. 2005;50(19):4681–94. 16177497 10.1088/0031-9155/50/19/017

[11] Létourneau D , Gulam M , Yan D , Oldham M , Wong JW . Evaluation of a 2D diode array for IMRT quality assurance. Radiother Oncol. 2004;70(2):199–206. 15028408 10.1016/j.radonc.2003.10.014

[12] Van Esch A , Depuydt T , Huyskens DP . The use of an aSi‐based EPID for routine absolute dosimetric pre‐treatment verification of dynamic IMRT fields. Radiother Oncol. 2004;71(2):223–34. 15110457 10.1016/j.radonc.2004.02.018

[13] Poppe B , Mehran P , Kollhoff R , Rubach A . [Use of a two‐dimensional ionization chamber array for quality assurance in medical linear accelerators] [in German]. Z Med Phys. 2002;13(2):115–22. 10.1078/0939-3889-00151 12868337

[14] Poppe B , Blechschmidt A , Djouguela A , et al. Two‐dimensional ionization chamber arrays for IMRT plan verification. Med Phys. 2006;33(4):1005–14. 16696477 10.1118/1.2179167

[15] Poppe B , Djouguela A , Blechschmidt A , Willborn K , Rühmann A , Harder D . Spatial resolution of 2D ionization chamber arrays for IMRT dose verification: single‐detector size and sampling step width. Phys Med Biol. 2007;52(10):2921–25. 17473360 10.1088/0031-9155/52/10/019

[16] Spezi E , Angelini A , Romani F , Ferri A . Characterization of a 2D ion chamber array for the verification of radiotherapy treatments. Phys Med Biol. 2005;50(14):3361–73. 16177515 10.1088/0031-9155/50/14/012

[17] Amerio S , Boriano A , Bourhaleb F , et al. Dosimetric characterization of a large area pixel‐segmented ionization chamber. Med Phys. 2004;31(2):414–20. 15000628 10.1118/1.1639992

[18] Herzen J , Todorovic M , Cremers F , et al. Dosimetric evaluation of a 2D pixel ionization chamber for implementation in clinical routine. Phys Med Biol. 2007;52(4):1197–208. 17264380 10.1088/0031-9155/52/4/023

[19] Wiezorek T , Banz N , Schwedas M , et al. Dosimetric quality assurance for intensity‐modulated radiotherapy feasibility study for a filmless approach. Strahlenther Onkol. 2005;181(7):468–74. 15995841 10.1007/s00066-005-1381-z

[20] Jursinic PA , Sharma R , Reuter J . MapCHECK used for rotational IMRT measurements: Step‐and‐shoot, Tomotherapy, RapidArc. Med Phys. 2010;37(6):2837–46. 20632595 10.1118/1.3431994

[21] Jursinic PA , Nelms BE . A 2‐D diode array and analysis software for verification of intensity modulated radiation therapy delivery. Med Phys. 2003;30(5):870–79. 12772995 10.1118/1.1567831

[22] Watts RJ . Evaluation of a diode detector array for use as a linear accelerator QC device. Med Phys. 1998;25(2):247–50. 9507488 10.1118/1.598188

[23] Bedford JL , Lee YK , Wai P , South CP , Warrington AP . Evaluation of the Delta4 phantom for IMRT and VMAT verification. Phys Med Biol. 2009;54(9):N167–76. 19384007 10.1088/0031-9155/54/9/N04

[24] Létourneau D , Publicover J , Kozelka J , Moseley DJ , Jaffray DA . Novel dosimetric phantom for quality assurance of volumetric modulated arc therapy. Med Phys. 2009;36(5):1813–21. 19544800 10.1118/1.3117563

[25] Petoukhova A , van Egmond J , Eenink M , Wiggenraad R , van Santvoort J . The ArcCHECK diode array for dosimetric verification of HybridArc. Phys Med Biol. 2011;56(16):5411–28. 21804180 10.1088/0031-9155/56/16/021

[26] Nelms BE , Opp D , Robinson J , et al. VMAT QA: measurement‐guided 4D dose reconstruction on a patient. Med Phys. 2012;39(7):4228–38. 22830756 10.1118/1.4729709

[27] Van Esch A , Clermont C , Devillers M , Iori M , Huyskens DP . On‐line quality assurance of rotational radiotherapy treatment delivery by means of a 2D ion chamber array and the Octavius phantom. Med Phys. 2007;34(10):3825–37. 17985628 10.1118/1.2777006

[28] Paliwal BR , Zaini M , McNutt T , Fairbanks EJ , Kitchen R . A consistency monitor for radiation therapy treatments. Med Phys. 1996;23(10):1805–07. 8946377 10.1118/1.597762

[29] Poppe B , Thieke C , Beyer D , et al. DAVID— a translucent multi‐wire transmission ionization chamber for in vivo verification of IMRT and conformal irradiation techniques. Phys Med Biol. 2006;51(5):1237–48. 16481690 10.1088/0031-9155/51/5/013

[30] Islam MK , Norrlinger BD , Smale JR , et al. An integral quality monitoring system for real‐time verification of intensity modulated radiation therapy. Med Phys. 2009;36(12):5420–28. 20095254 10.1118/1.3250859

[31] Venkataraman S , Malkoske KE , Jensen M , Nakonechny KD , Asuni G , McCurdy BM . The influence of a novel transmission detector on 6 MV x‐ray beam characteristics. Phys Med Biol. 2009;54(10):3173–83. 19420428 10.1088/0031-9155/54/10/014

[32] Asuni G , Jensen J , McCurdy BM . A Monte Carlo investigation of contaminant electrons due to a novel in vivo transmission detector. Phys Med Biol. 2011;56(4):1207–23. 21285480 10.1088/0031-9155/56/4/020

[33] Poppe B , Looe HK , Chofor N , Rühmann A , Harder D , Willborn KC . Clinical performance of a transmission detector array for the permanent supervision of IMRT deliveries. Radiother Oncol. 2010;95(2):158–65. 20138379 10.1016/j.radonc.2009.12.041

[34] Delta4 Discover. Sweden: ScandiDos Available from: http://scandidos.com/home/solutions/at-treatment-qa/deltasup4sup-discover. Accessed 21/01/2015.

[35] Online Treatment Monitoring dolphin. Schwarzenbruck, Germany: IBA Dosimetry Available from: http://www.iba-dosimetry.com/complete-solutions/sbrt-online-treatment-monitoring Accessed 21/01/2015.

[36] Wong J , Fuduli I , Carolan M , et al. Characterization of a novel two dimensional diode array the “magic plate” as a radiation detector for radiation therapy treatment. Med Phys. 2012;39(5):2544–58. 22559625 10.1118/1.3700234

[37] Alrowaili ZA , Lerch MLF , Carolan M , et al. 2D mapping of the MV photon fluence and 3D dose reconstruction in real time for quality assurance during radiotherapy treatment. J Instrument. 2015;10(09):P09019.

[38] Devic S , Seuntjens J , Abdel‐Rahman W , et al. Accurate skin dose measurements using radiochromic film in clinical applications. Med Phys. 2006;33(4):1116–24. 16696489 10.1118/1.2179169

[39] Kim S , Liu CR , Zhu TC , Palta JR . Photon beam skin dose analyses for different clinical setups. Med Phys. 1998;25(6):860–66. 9650173 10.1118/1.598261

[40] McParland BJ . The effects of a universal wedge and beam obliquity upon the central axis dose buildup for 6‐MV x rays. Med Phys. 1991;18(4):740–43. 1921879 10.1118/1.596733

[41] Lamb A and Blake S . Investigation and modelling of the surface dose from linear accelerator produced 6 and 10 MV photon beams. Phys Med Biol. 1998;43(5):1133–46. 9623645 10.1088/0031-9155/43/5/006

[42] Li Z and Klein EE . Surface and peripheral doses of dynamic and physical wedges. Int J Radiat Oncol Biol Phys. 1997;37(4):921–25. 9128970 10.1016/s0360-3016(96)00610-4

[43] Aldosari A , Espinoza A , Robinson D , et al. Characterization of an innovative p‐type epitaxial diode for dosimetry in modern external beam radiotherapy. Nucl Sci, IEEE Transactions on. 2013;60(6):4705–12.

[44] Nilsson B and Montelius A . Fluence perturbation in photon beams under nonequilibrium conditions. Med Phys. 1986;13(2):191–95. 3702815 10.1118/1.595895

[45] Rubach A , Conrad F , Bichsel H . Dose build‐up curves for cobalt‐60 irradiation: a systematic error occurring with pancake chamber measurements. Phys Med Biol. 1986;31(4):441–48. 3737683 10.1088/0031-9155/31/4/009

[46] Velkley D , Manson D , Purdy J , Oliver Jr G . Build‐up region of megavoltage photon radiation sources. Med Phys. 1975;2(1):14–19. 805358 10.1118/1.594158

[47] Gerbi BJ and Khan FM . Measurement of dose in the buildup region using fixed‐separation plane‐parallel ionization chambers. Med Phys. 1990;17(1):17–26. 2106611 10.1118/1.596522

[48] Mellenberg DE Jr . Determination of build‐up region over‐response corrections for a Markus‐type chamber. Med Phys. 1990;17(6):1041–44. 2280733 10.1118/1.596579

[49] Rawlinson JA , Arlen D , Newcombe D . Design of parallel plate ion chambers for buildup measurements in megavoltage photon beams. Med Phys. 1992;19(3):641–48. 1508103 10.1118/1.596896

[50] Chen F , Gupta R , Metcalfe P . Intensity modulated radiation therapy (IMRT) surface dose measurements using a PTW advanced Markus chamber. Australas Phys Eng Sci Med. 2010;33(1):23–34. 20237890 10.1007/s13246-010-0004-x

