# Supplementary figures and images for: Beam perturbation characteristics of a 2D transmission silicon diode array, Magic Plate
Source: J Appl Clin Med Phys. 2016 Mar 8;17(2):85–98. doi: 10.1120/jacmp.v17i2.5932 (PMC5874939; doi:10.1120/jacmp.v17i2.5932)

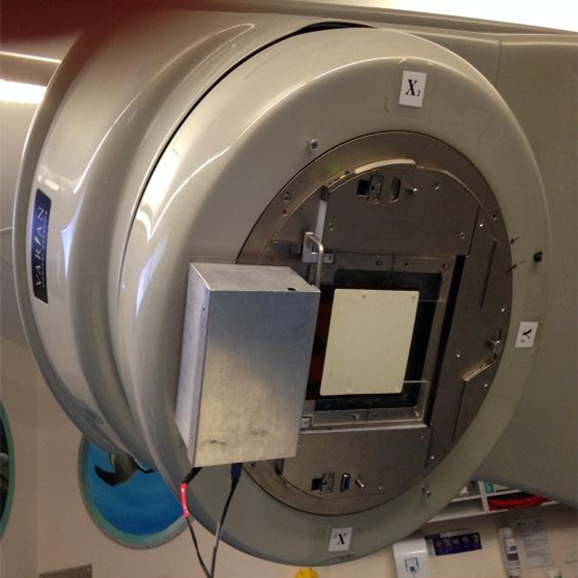

Supplement: Supplementary file 1 — Supplementary Material Files [file ACM2-17-85-s001.png]

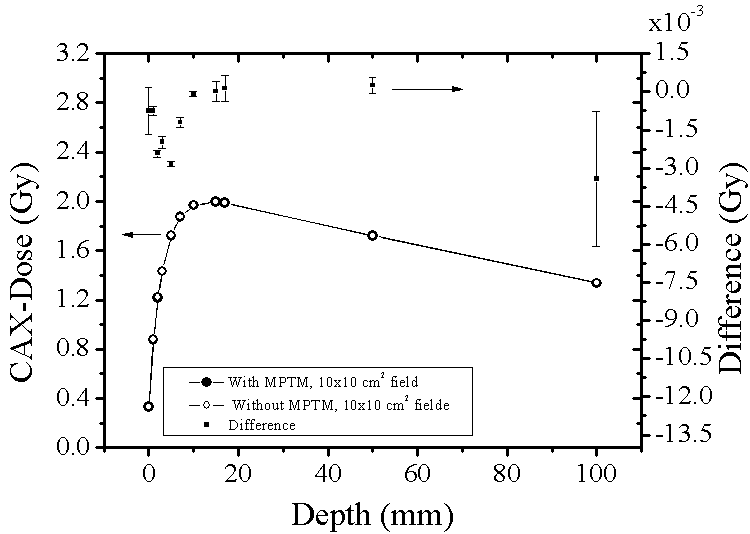

Supplement: Supplementary file 2 — Supplementary Material Files [file ACM2-17-85-s002.png]

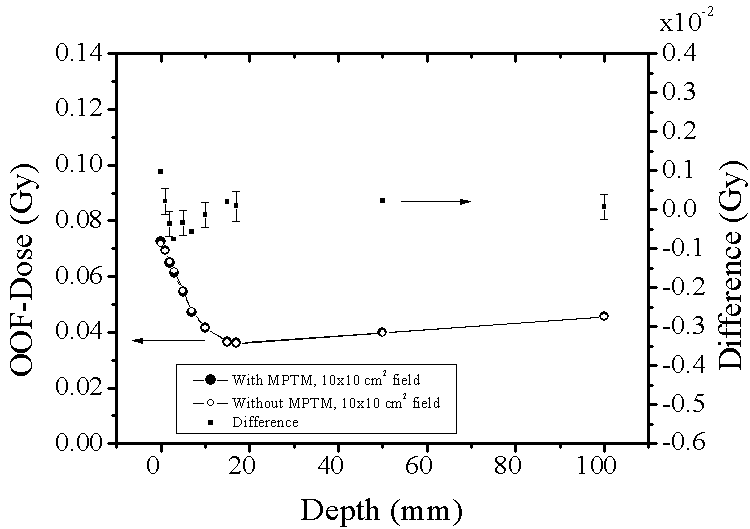

Supplement: Supplementary file 3 — Supplementary Material Files [file ACM2-17-85-s003.png]

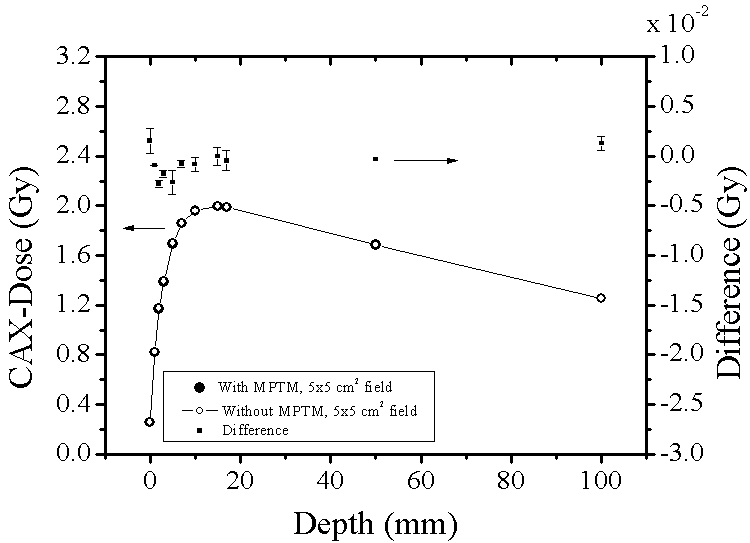

Supplement: Supplementary file 4 — Supplementary Material Files [file ACM2-17-85-s004.png]

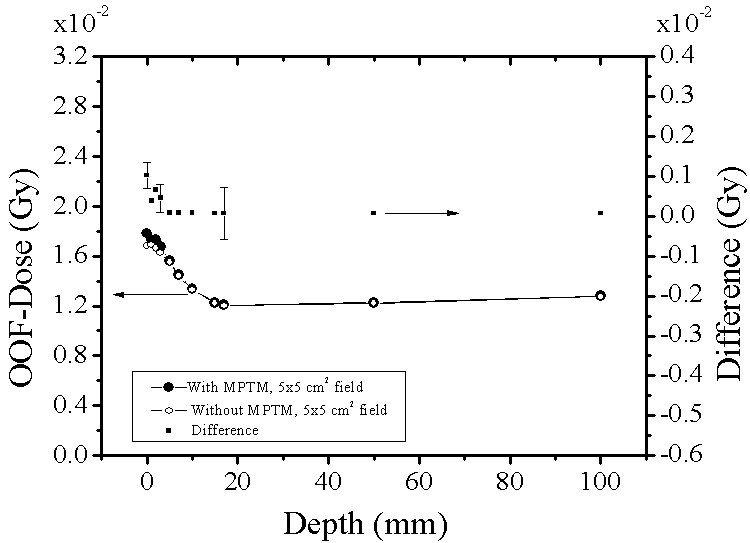

Supplement: Supplementary file 5 — Supplementary Material Files [file ACM2-17-85-s005.png]

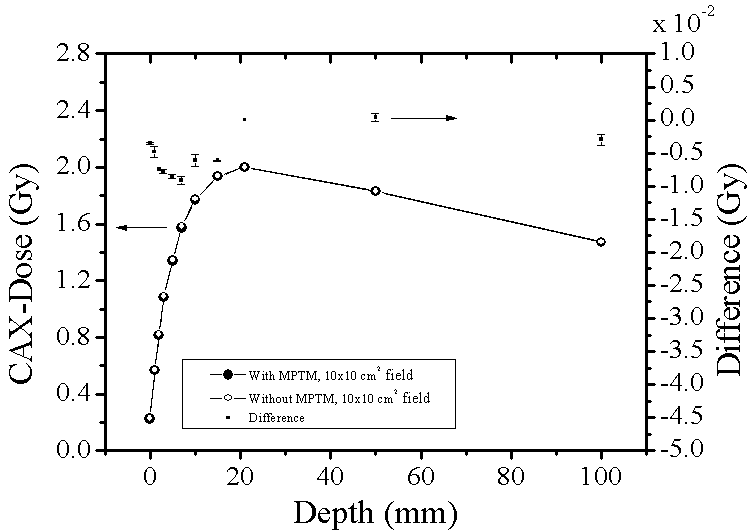

Supplement: Supplementary file 6 — Supplementary Material Files [file ACM2-17-85-s006.png]

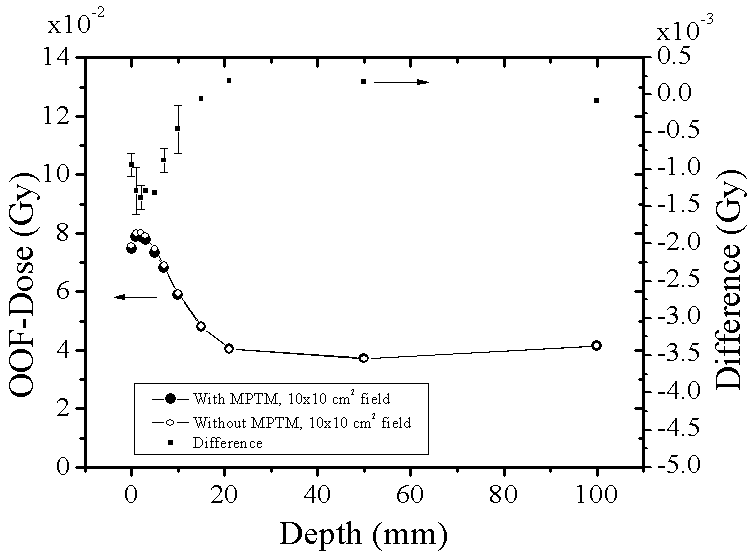

Supplement: Supplementary file 7 — Supplementary Material Files [file ACM2-17-85-s007.png]

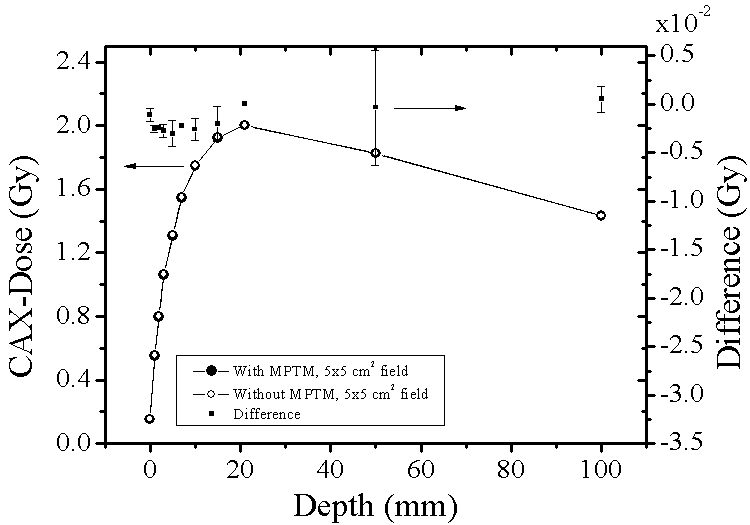

Supplement: Supplementary file 8 — Supplementary Material Files [file ACM2-17-85-s008.png]

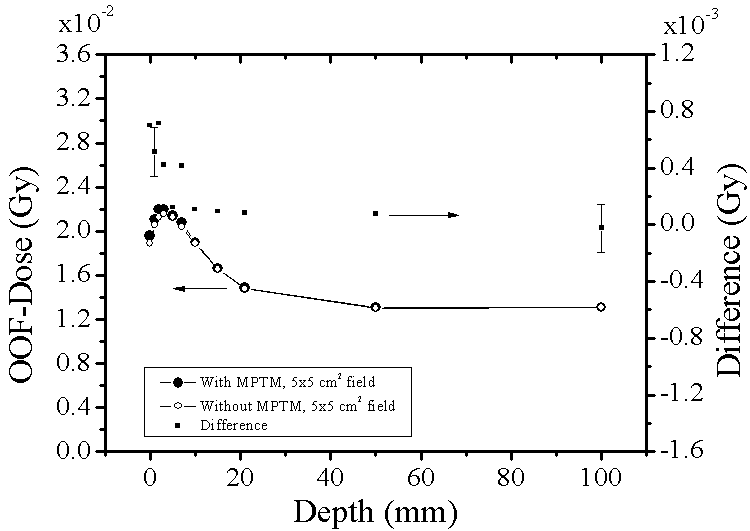

Supplement: Supplementary file 9 — Supplementary Material Files [file ACM2-17-85-s009.png]

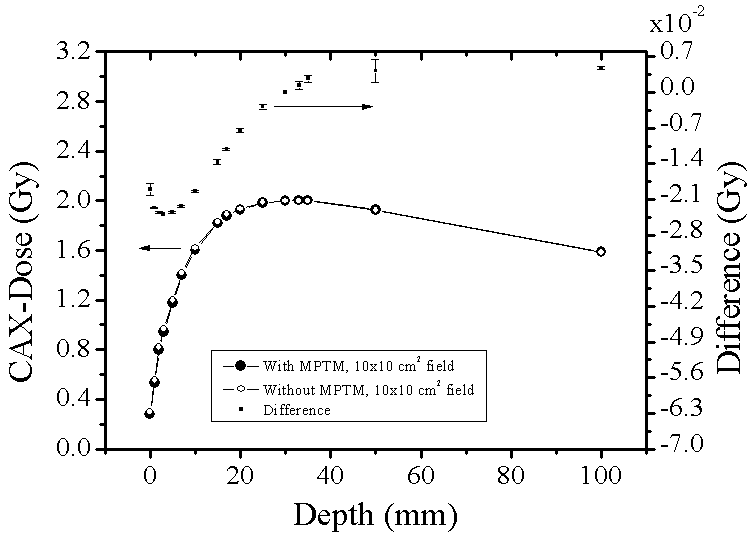

Supplement: Supplementary file 10 — Supplementary Material Files [file ACM2-17-85-s010.png]

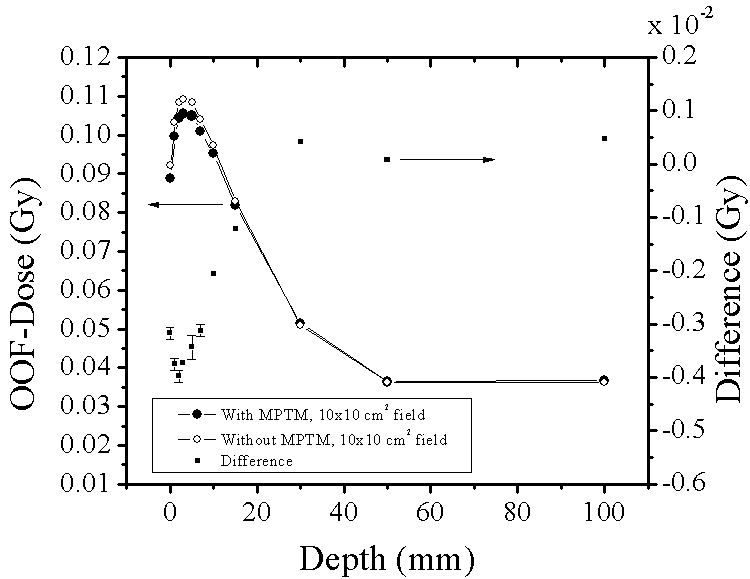

Supplement: Supplementary file 11 — Supplementary Material Files [file ACM2-17-85-s011.png]

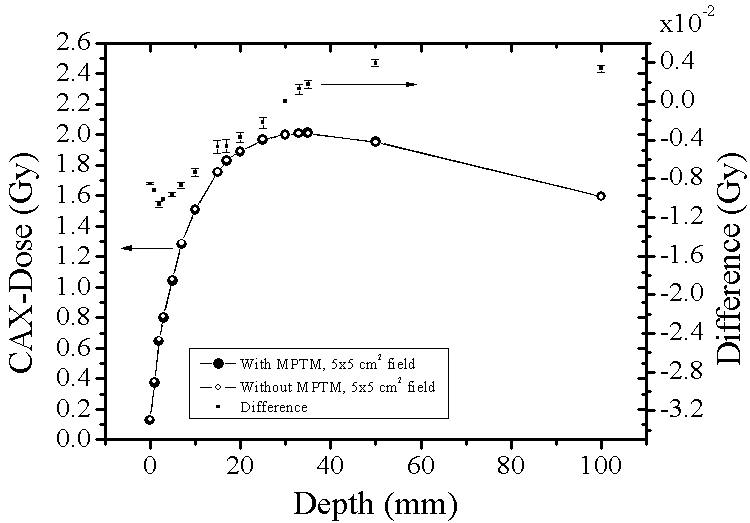

Supplement: Supplementary file 12 — Supplementary Material Files [file ACM2-17-85-s012.png]

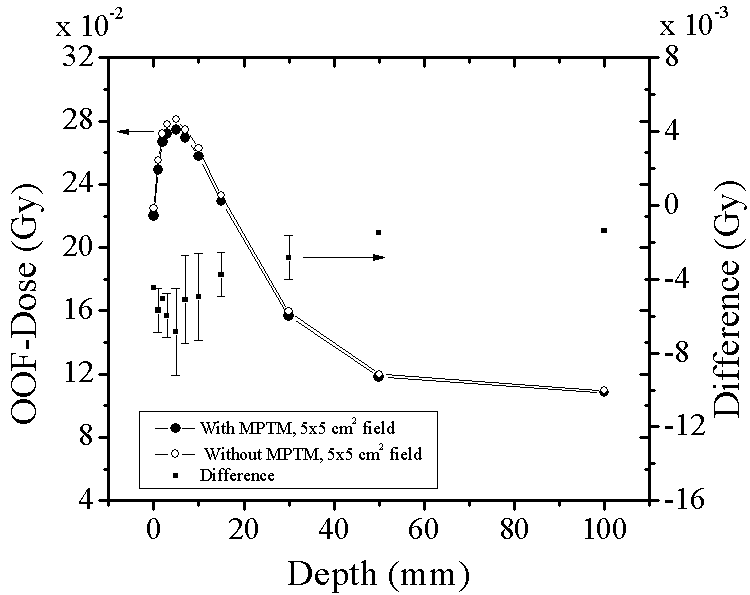

Supplement: Supplementary file 13 — Supplementary Material Files [file ACM2-17-85-s013.png]

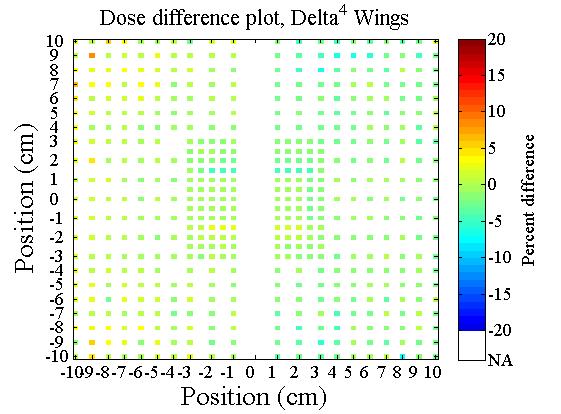

Supplement: Supplementary file 14 — Supplementary Material Files [file ACM2-17-85-s014.jpg]

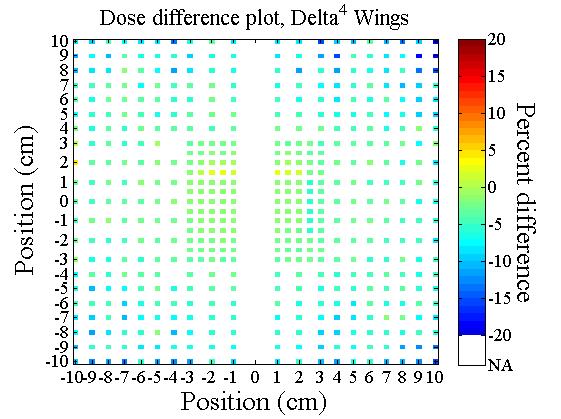

Supplement: Supplementary file 15 — Supplementary Material Files [file ACM2-17-85-s015.jpg]

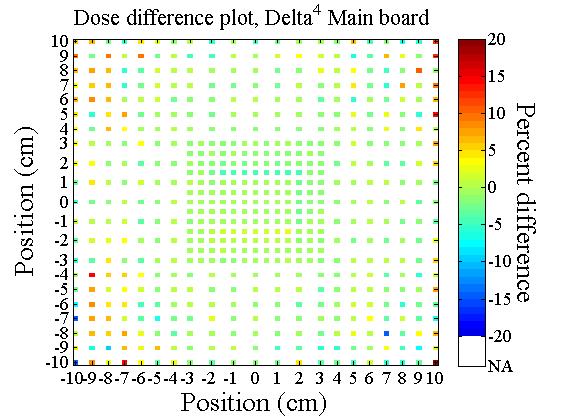

Supplement: Supplementary file 16 — Supplementary Material Files [file ACM2-17-85-s016.jpg]

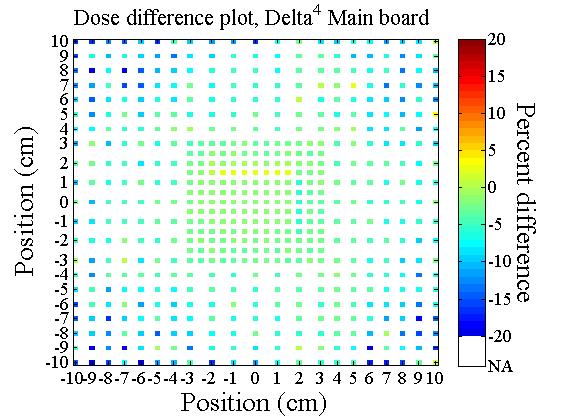

Supplement: Supplementary file 17 — Supplementary Material Files [file ACM2-17-85-s017.jpg]

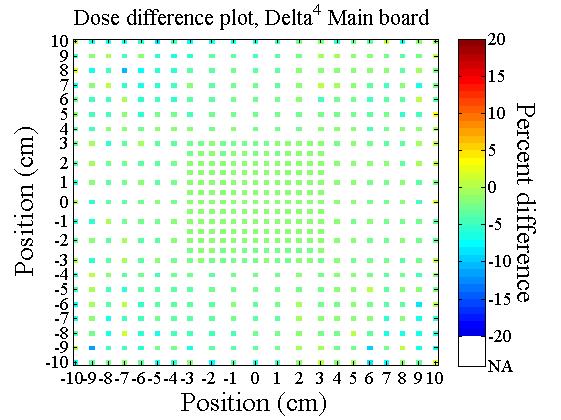

Supplement: Supplementary file 18 — Supplementary Material Files [file ACM2-17-85-s018.jpg]

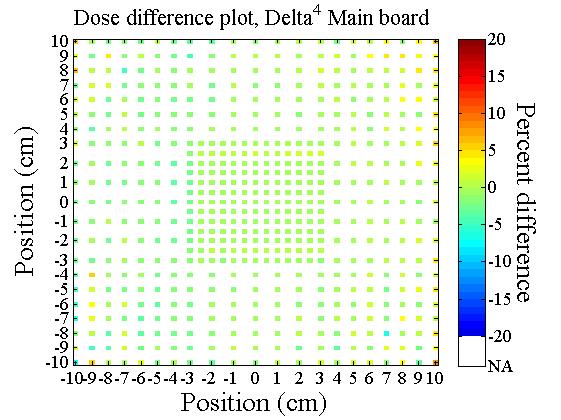

Supplement: Supplementary file 19 — Supplementary Material Files [file ACM2-17-85-s019.jpg]

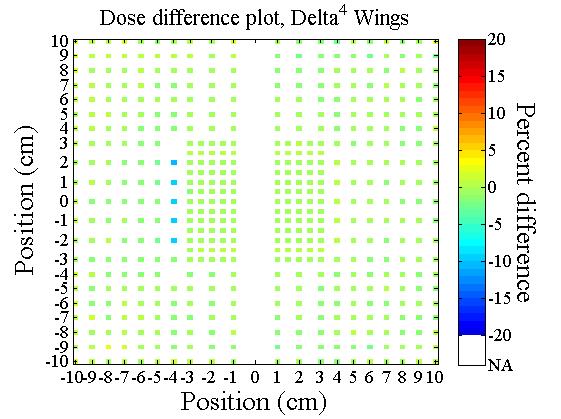

Supplement: Supplementary file 20 — Supplementary Material Files [file ACM2-17-85-s020.jpg]

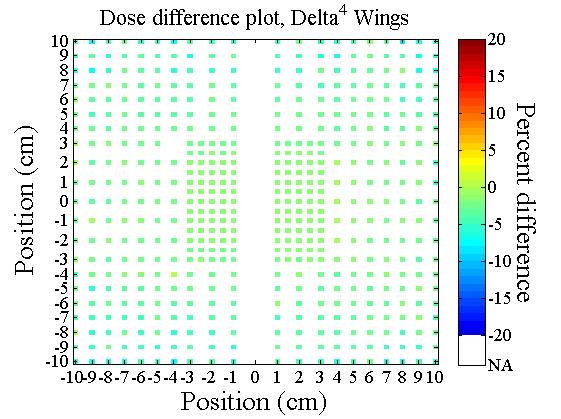

Supplement: Supplementary file 21 — Supplementary Material Files [file ACM2-17-85-s021.jpg]

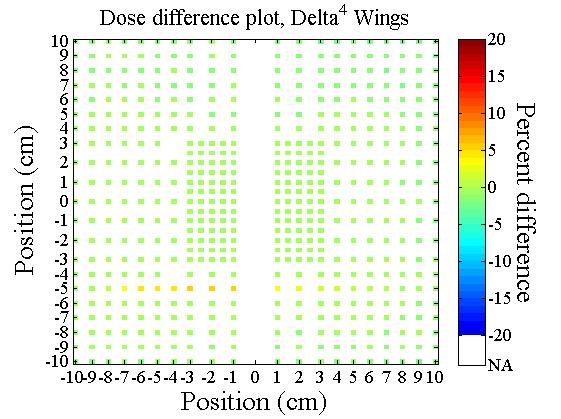

Supplement: Supplementary file 22 — Supplementary Material Files [file ACM2-17-85-s022.jpg]

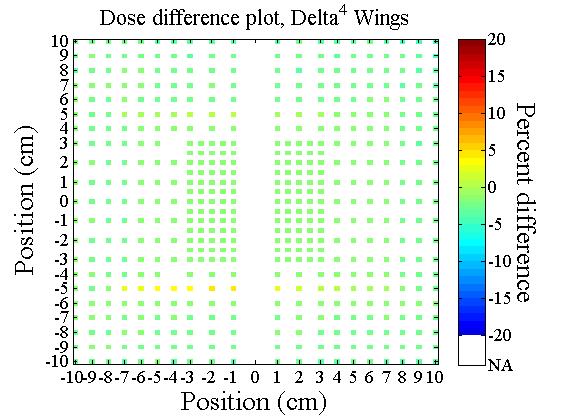

Supplement: Supplementary file 23 — Supplementary Material Files [file ACM2-17-85-s023.jpg]

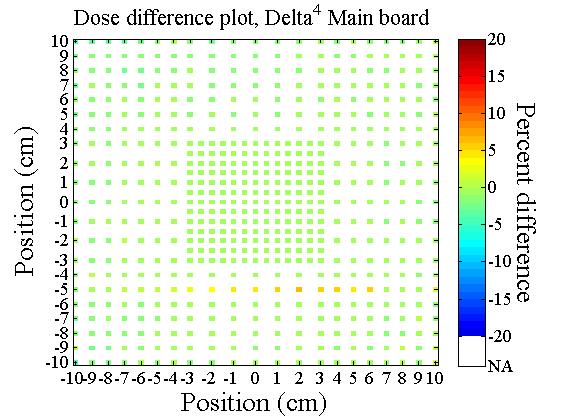

Supplement: Supplementary file 24 — Supplementary Material Files [file ACM2-17-85-s024.jpg]

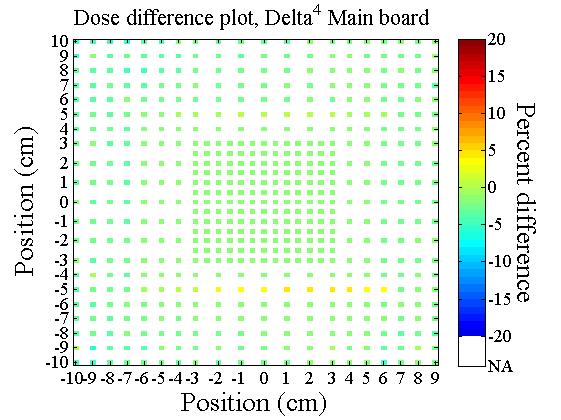

Supplement: Supplementary file 25 — Supplementary Material Files [file ACM2-17-85-s025.jpg]

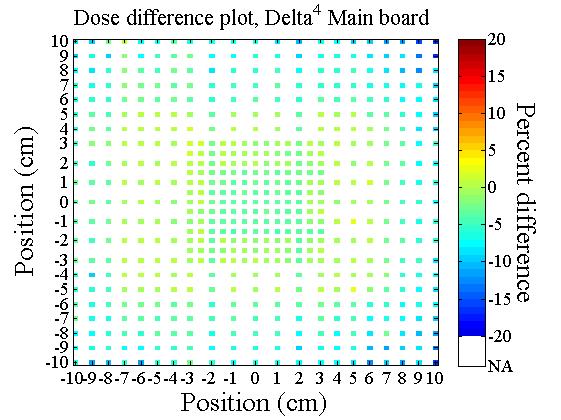

Supplement: Supplementary file 26 — Supplementary Material Files [file ACM2-17-85-s026.jpg]

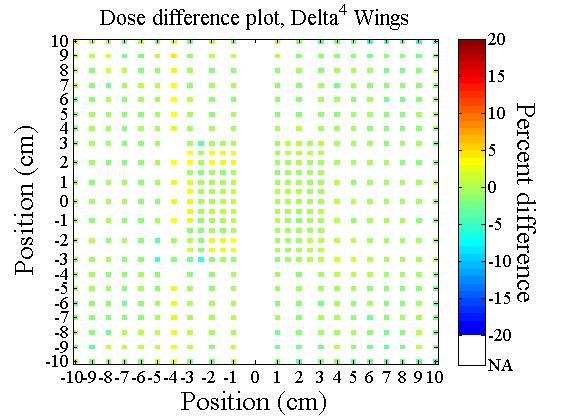

Supplement: Supplementary file 27 — Supplementary Material Files [file ACM2-17-85-s027.jpg]

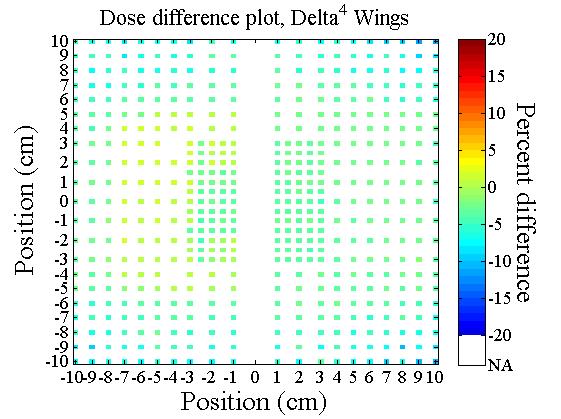

Supplement: Supplementary file 28 — Supplementary Material Files [file ACM2-17-85-s028.jpg]

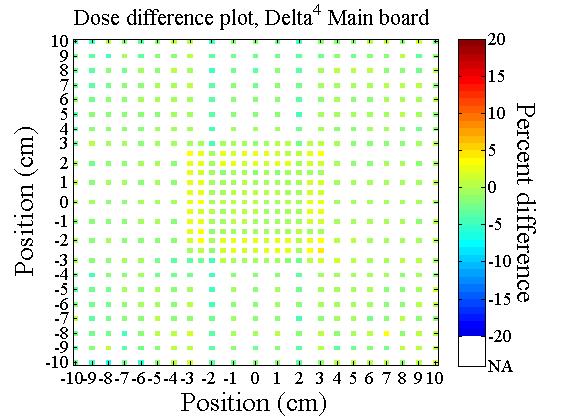

Supplement: Supplementary file 29 — Supplementary Material Files [file ACM2-17-85-s029.jpg]

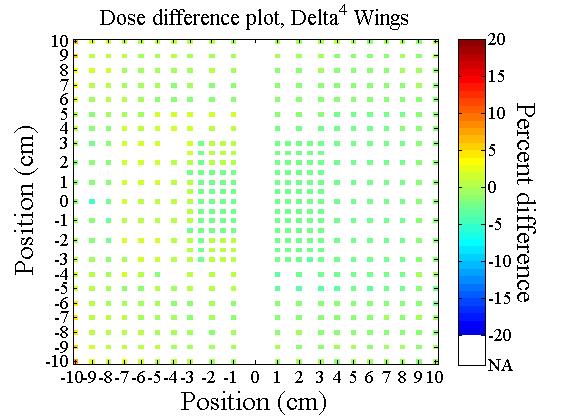

Supplement: Supplementary file 30 — Supplementary Material Files [file ACM2-17-85-s030.jpg]

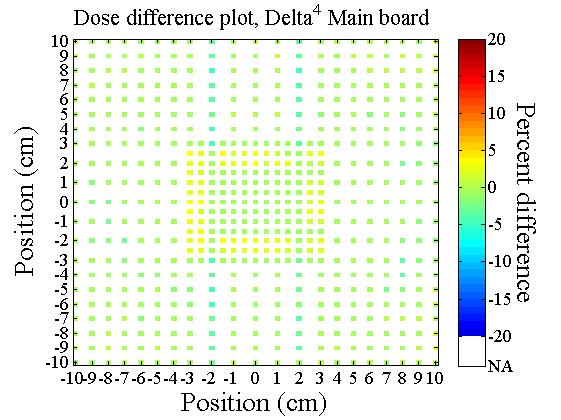

Supplement: Supplementary file 31 — Supplementary Material Files [file ACM2-17-85-s031.jpg]

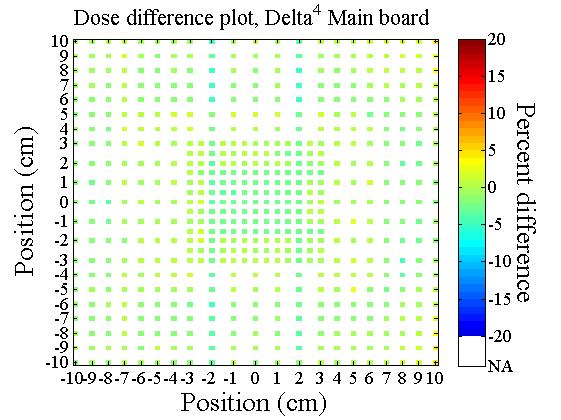

Supplement: Supplementary file 32 — Supplementary Material Files [file ACM2-17-85-s032.jpg]

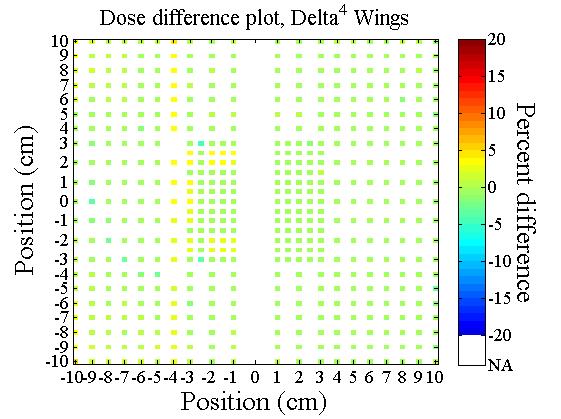

Supplement: Supplementary file 33 — Supplementary Material Files [file ACM2-17-85-s033.jpg]

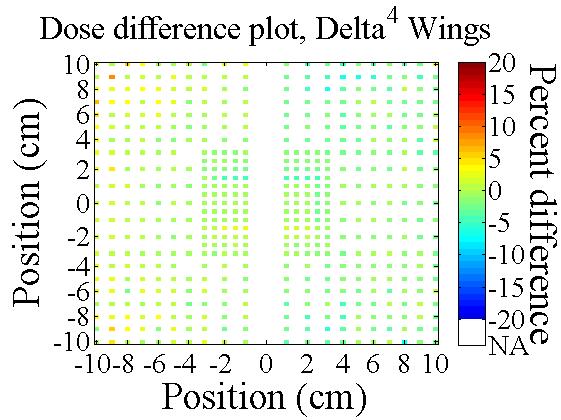

Supplement: Supplementary file 34 — Supplementary Material Files [file ACM2-17-85-s034.jpg]

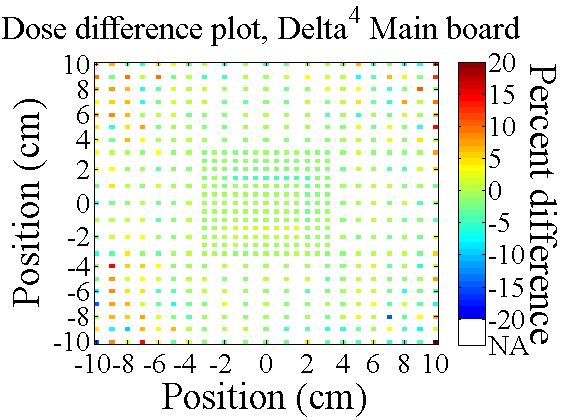

Supplement: Supplementary file 35 — Supplementary Material Files [file ACM2-17-85-s035.jpg]

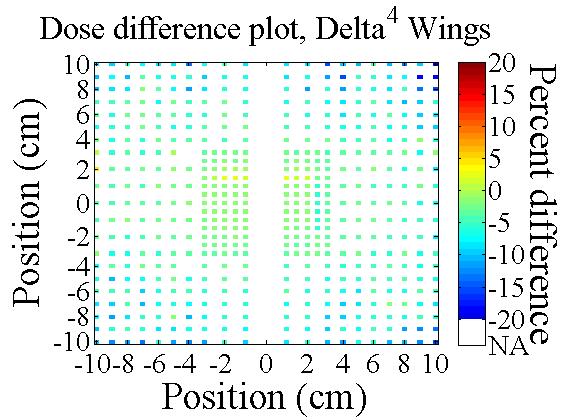

Supplement: Supplementary file 36 — Supplementary Material Files [file ACM2-17-85-s036.jpg]

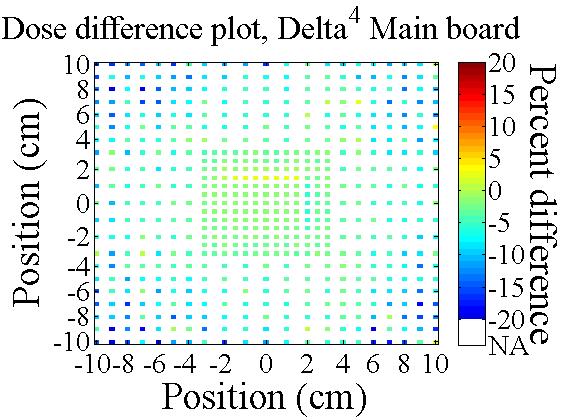

Supplement: Supplementary file 37 — Supplementary Material Files [file ACM2-17-85-s037.jpg]

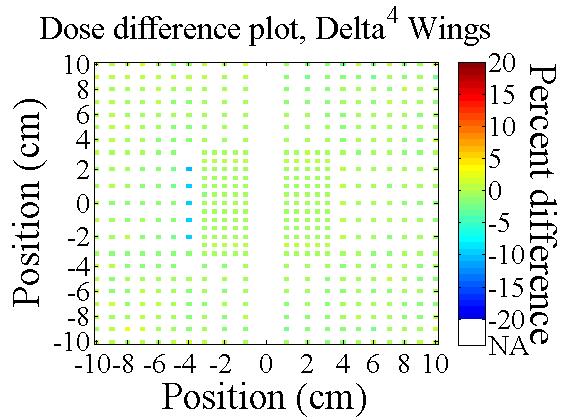

Supplement: Supplementary file 38 — Supplementary Material Files [file ACM2-17-85-s038.jpg]

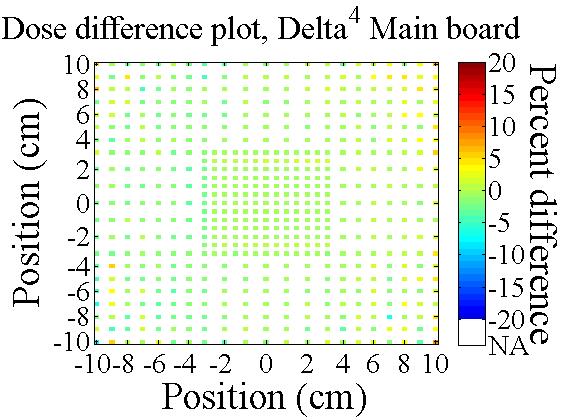

Supplement: Supplementary file 39 — Supplementary Material Files [file ACM2-17-85-s039.jpg]

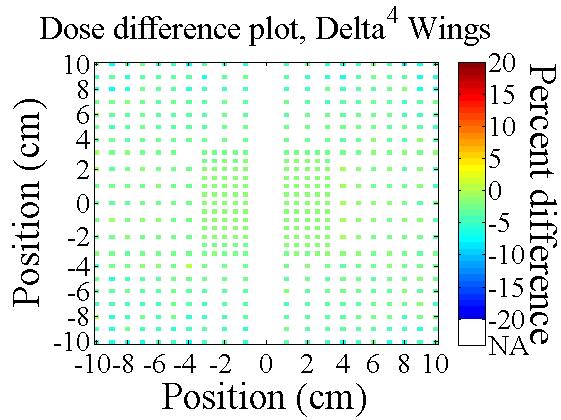

Supplement: Supplementary file 40 — Supplementary Material Files [file ACM2-17-85-s040.jpg]

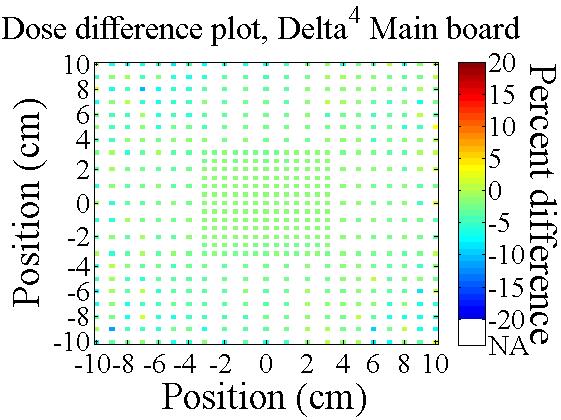

Supplement: Supplementary file 41 — Supplementary Material Files [file ACM2-17-85-s041.jpg]

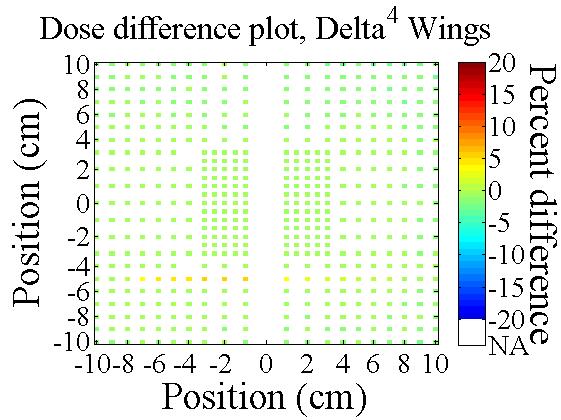

Supplement: Supplementary file 42 — Supplementary Material Files [file ACM2-17-85-s042.jpg]

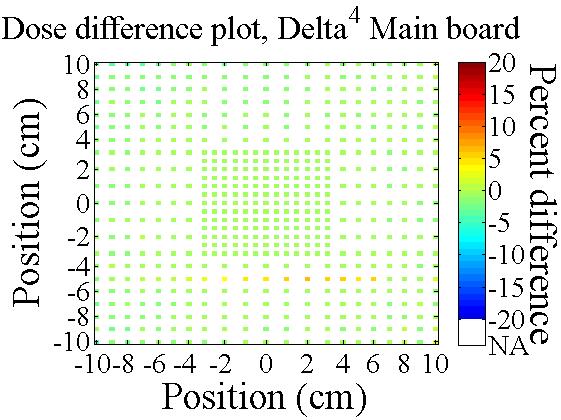

Supplement: Supplementary file 43 — Supplementary Material Files [file ACM2-17-85-s043.jpg]

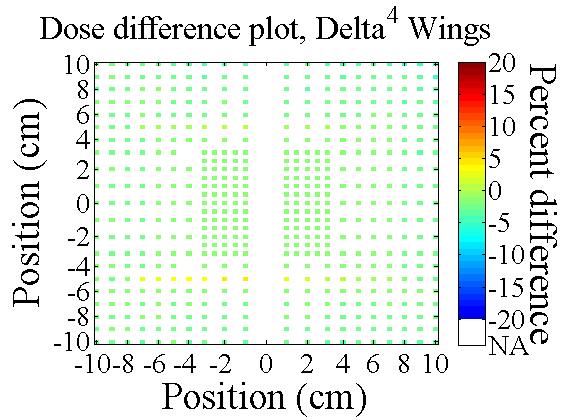

Supplement: Supplementary file 44 — Supplementary Material Files [file ACM2-17-85-s044.jpg]

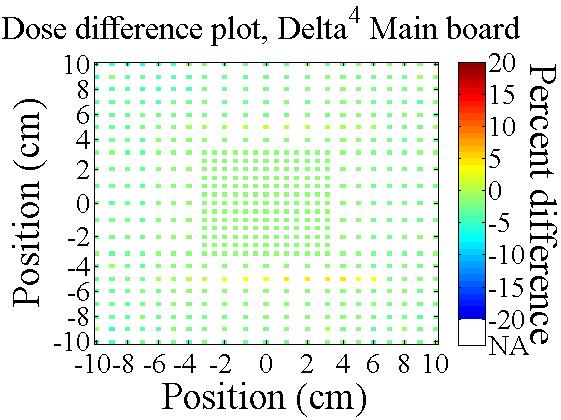

Supplement: Supplementary file 45 — Supplementary Material Files [file ACM2-17-85-s045.jpg]

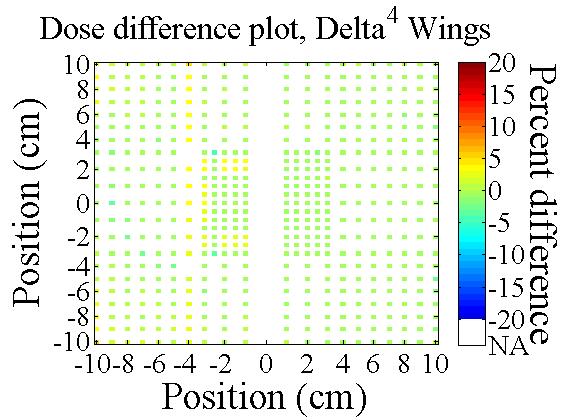

Supplement: Supplementary file 46 — Supplementary Material Files [file ACM2-17-85-s046.jpg]

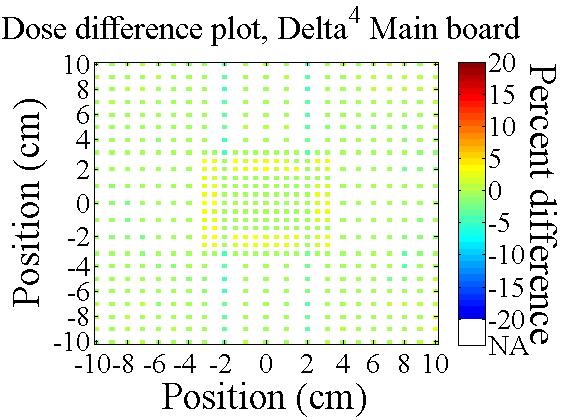

Supplement: Supplementary file 47 — Supplementary Material Files [file ACM2-17-85-s047.jpg]

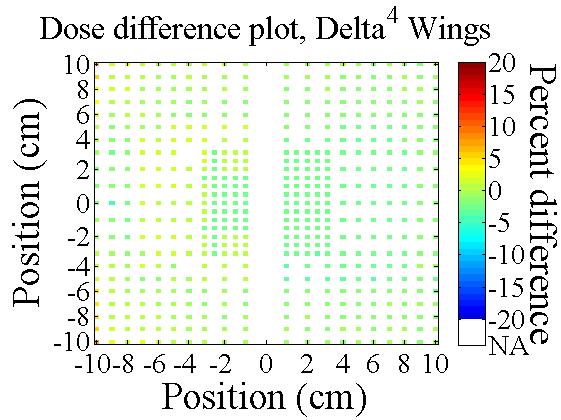

Supplement: Supplementary file 48 — Supplementary Material Files [file ACM2-17-85-s048.jpg]

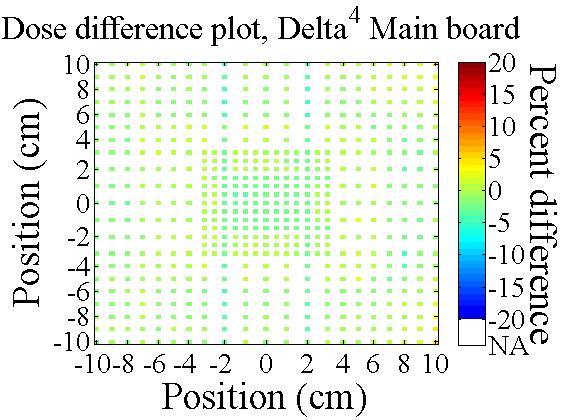

Supplement: Supplementary file 49 — Supplementary Material Files [file ACM2-17-85-s049.jpg]

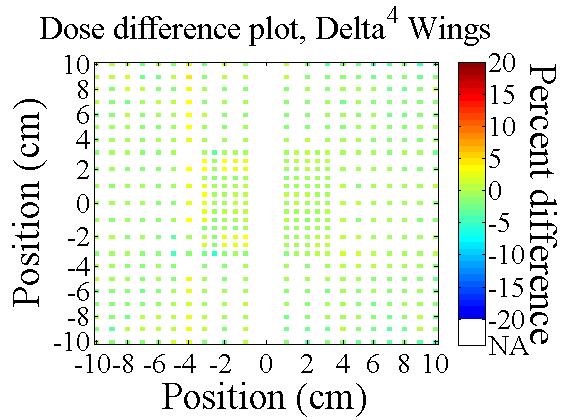

Supplement: Supplementary file 50 — Supplementary Material Files [file ACM2-17-85-s050.jpg]

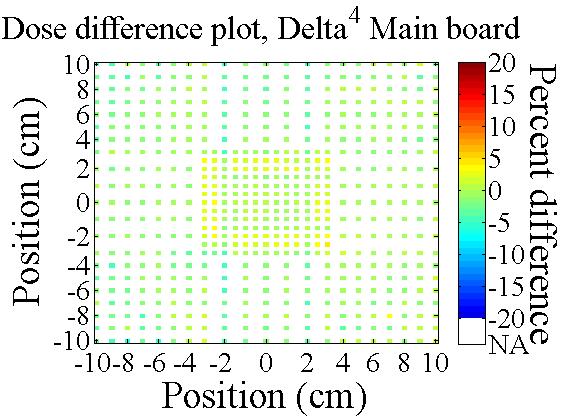

Supplement: Supplementary file 51 — Supplementary Material Files [file ACM2-17-85-s051.jpg]

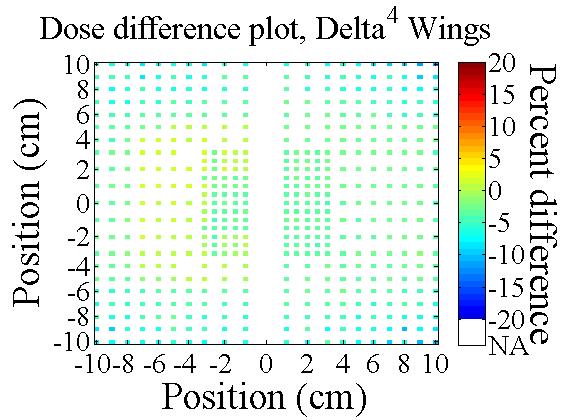

Supplement: Supplementary file 52 — Supplementary Material Files [file ACM2-17-85-s052.jpg]

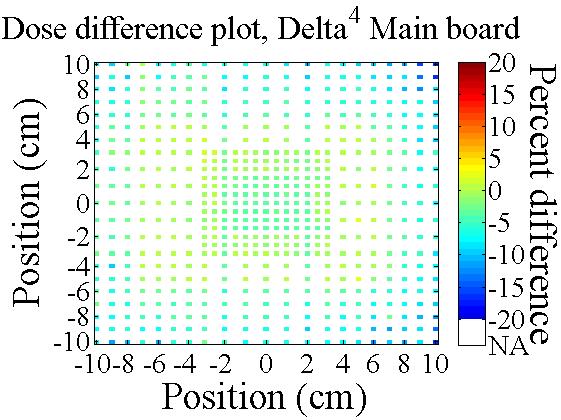

Supplement: Supplementary file 53 — Supplementary Material Files [file ACM2-17-85-s053.jpg]
